# A call for comparative effectiveness research to learn whether routine clinical care decisions can protect from dementia and cognitive decline

**DOI:** 10.1186/s13195-016-0200-3

**Published:** 2016-08-20

**Authors:** Penny A. Dacks, Joshua J. Armstrong, Stephen K. Brannan, Aaron J. Carman, Allan M. Green, M. Sue Kirkman, Lawrence R. Krakoff, Lewis H. Kuller, Lenore J. Launer, Simon Lovestone, Elizabeth Merikle, Peter J. Neumann, Kenneth Rockwood, Diana W. Shineman, Richard G. Stefanacci, Priscilla Velentgas, Anand Viswanathan, Rachel A. Whitmer, Jeff D. Williamson, Howard M. Fillit

**Affiliations:** 1 Alzheimer’s Drug Discovery Foundation, 57 West 57th St. Suite 901, New York, NY 10019 USA; 2 Geriatric Medicine Research Unit, Department of Medicine, Dalhousie University, Halifax, NS Canada; 3 Takeda Pharmaceuticals, Deerfield, IL USA; 4 Attorney at Law, Cambridge, MA USA; 5 Department of Medicine, Division of Endocrinology and Metabolism, University of North Carolina School of Medicine, Chapel Hill, NC USA; 6 Icahn School of Medicine at Mount Sinai, New York, NY USA; 7 Department of Epidemiology, Graduate School of Public Health, University of Pittsburgh, Pittsburgh, PA USA; 8 Intramural Research Program, National Institute on Aging, NIH, Bethesda, MD USA; 9 Department of Psychiatry, University of Oxford, Oxford, UK; 10 Center for the Evaluation of Value and Risk in Health, Institute for Clinical Research and Health Policy Studies, Tufts Medical Center, Boston, MA USA; 11 DGI Clinical, Halifax, NS Canada; 12 Nova Scotia Health Authority, Halifax, NS Canada; 13 Thomas Jefferson College of Population Health, The Access Group, Philadelphia, PA USA; 14 Scientific Affairs, Quintiles Real World Late Phase Research, Cambridge, MA USA; 15 Representative for the American Heart Association; Hemorrhagic Stroke Research Program, Department of Neurology, Massachusetts General Hospital Stroke Research Center, Harvard Medical School, Boston, MA USA; 16 Kaiser Permanente Division of Research, Population Science and Brain Aging, Oakland, CA USA; 17 Wake Forest University School of Medicine, Winston-Salem, NC USA

**Keywords:** Comparative effectiveness, Dementia, Alzheimer’s, Prevention, Cognitive decline, Cognitive aging, Comorbidity, Repurposing, Hypertension, Diabetes

## Abstract

Common diseases like diabetes, hypertension, and atrial fibrillation are probable risk factors for dementia, suggesting that their treatments may influence the risk and rate of cognitive and functional decline. Moreover, specific therapies and medications may affect long-term brain health through mechanisms that are independent of their primary indication. While surgery, benzodiazepines, and anti-cholinergic drugs may accelerate decline or even raise the risk of dementia, other medications act directly on the brain to potentially slow the pathology that underlies Alzheimer’s and other dementia. In other words, the functional and cognitive decline in vulnerable patients may be influenced by the choice of treatments for other medical conditions. Despite the importance of these questions, very little research is available. The Alzheimer’s Drug Discovery Foundation convened an advisory panel to discuss the existing evidence and to recommend strategies to accelerate the development of comparative effectiveness research on how choices in the clinical care of common chronic diseases may protect from cognitive decline and dementia.

## Background

Cognitive impairment diagnosed as dementia, mild cognitive impairment (MCI), and/or mild neurocognitive disorder (mNCD) represents one of the most feared conditions in the United States and one of the most common reasons to enter a nursing home. In North America alone, the financial cost of dementia was estimated at $270 billion for 2015 with $61 billion in direct medical costs [1]. The burden from MCI is more difficult to quantify [2] but profound effects on quality of life, productivity, and health are likely [3, 4]. Cognitive aging itself, while not a diagnosed medical condition, can influence quality of life and can be protected against through a variety of steps outlined in a recent Institute of Medicine report [5]. The consequences of cognitive decline pervade other aspects of health. For example, patients with cognitive impairment are less likely to comply with prescribed treatments and more likely to require hospitalization or experience treatment-related adverse events.

The factors that contribute to the risk and progression of MCI and dementia are varied and uncertain even for Alzheimer’s disease, the most extensively researched cause of dementia [6]. Several different trajectories appear to lead to the clinical phenotype of Alzheimer’s disease, with distinct molecular causes and risk factors as well as different patterns of progression [7] as reflected by the inability to generate a single risk prediction model for population-based settings [8]. Combinations of neuropathology are common and probably contribute synergistically to cognitive impairment (e.g. [9]).

For many patients, the comorbidities and the corresponding medical care may contribute to their long-term risk of cognitive and functional decline. These factors, in turn, may provide opportunities for precision medicine to tailor patient treatment according to their risk profile.

In 2015, the Alzheimer’s Drug Discovery Foundation convened an advisory panel on “The Prevention of Dementia and Mild Cognitive Impairment as Variables to Consider In the Comparative Effectiveness of Treatments for Common Chronic Conditions.” The panel included representatives of the American Diabetes Association and the American Heart Association as well as experts from industry, academic and government research institutes, and the Patient-Centered Outcomes Research Institute (PCORI).

The consensus perspective was that clinical care of chronic diseases and comorbidities can likely influence cognitive decline, particularly in high-risk patients, but the existing evidence base for specific treatment choices is weak and conflicted. The meeting discussions were formulated into five recommendations for researchers, clinicians, and other stakeholders to advance research on how clinical treatment options may influence long-term cognitive decline. The views expressed are those of the authors and not their organizations.

### Prevalent diseases are risk factors for cognitive decline and dementia

Several major chronic diseases are risk factors for dementia, linked by both epidemiology and biological rationale, suggesting that their clinical management might influence cognitive decline.

Hypertension in mid-life associates with a higher risk of dementia (reviewed in [10]). In the SYST-EUR trial, nitrendipine reduced the risk of Alzheimer’s or vascular dementia by about 50 % [11]. In the PROGRESS trial, an angiotensin converting enzyme (ACE) inhibitor reduced the risk of cognitive decline by 19 % largely because of a reduced risk of recurrent stroke [12]. Other trials have not reported such benefits [13], likely because of differences in study design and patient population (reviewed in [10]). In some cases, the null result might have been due to outcome ascertainment bias such as in the SHEP trial, in which patients assigned to placebo were 24–60 % more likely to miss their outcome assessment [14].

Atrial fibrillation has also been associated with dementia. This relationship is independent of stroke (reviewed by [15]) and stronger with longer durations of atrial fibrillation [16]. A causative relationship is possible through repetitive microemboli or microbleeds but research is needed to show whether choices in the treatment of atrial fibrillation can influence dementia risk [15].

Type 2 diabetes mellitus (T2DM) is also associated with a higher risk of Alzheimer’s (reviewed in [17, 18]). Insulin resistance and glucose dysmetabolism in the brain may directly drive Alzheimer’s neuropathology and increase the risk of cerebral infarcts that can in turn contribute to the manifestation of dementia (reviewed in [17]). Whether the clinical management of diabetes can influence cognitive decline is not yet certain. In the ACCORD-MIND trial, intensive versus standard glycemic control failed to protect against cognitive decline over 40 months but did result in a higher total brain volume [19].

Other diseases linked to cognitive decline include sleep-disordered breathing [20], heart failure [21], and chronic obstructive pulmonary disease [22]. More research is needed to establish whether these relationships are causal and, most importantly, if and how their clinical management can influence cognitive decline. Frailty and the accumulation of health deficits with old age [23] have also been linked to a higher risk of dementia, serving as an important reminder that biological vulnerabilities caused by aging underlie a suite of serious and common age-related ailments.

### Specific medical treatments may influence the risk of cognitive decline

Several common medical treatments are suspected to increase the risk of long-term cognitive decline. Cumulative anti-cholinergic drug burden has been associated with a dose-dependent increase of incident dementia as high as 54 % [24]. Similar risks have been reported for benzodiazepines and proton pump inhibitors [25]. Surgery can cause delirium or cognitive dysfunction in vulnerable patients which, in turn, associates with a higher risk of poor functional outcomes including dementia (reviewed in [26]). An ongoing controversy is whether surgery can cause persistent cognitive decline or simply unmask an underlying neurodegenerative illness (e.g. [27]). Even if the latter is true, earlier manifestation of progressive dementia is a significant concern.

While some treatments may increase the risk or the rate of cognitive decline, others may reduce it. Roughly 2 million cases of Alzheimer’s disease have been attributed to physical inactivity, smoking, and mid-life obesity [28], suggesting that lifestyle modifications for cardiometabolic disease may profoundly protect the brain (reviewed in [29]). In clinical trials, cognitive function in the elderly has been improved through diet [30] or a multi-faceted intervention of lifestyle, diet, and vascular risk management [31]. These studies must be followed with longer and larger studies to evaluate effects on dementia risk.

There is intense interest in the question of whether specific drugs can be repurposed to treat or prevent neurodegenerative disease. Table 1 lists clinical trials that are underway to test whether specific drugs might reduce the risk or slow the progression of dementia or MCI through direct effects in the brain that are independent of their approved indications for treating high blood pressure or blood glucose. If these drugs do indeed slow neurodegeneration, a promising strategy to reduce dementia risk would be to manage hypertension or diabetes in high-risk patients with a clinically appropriate drug that has additional beneficial effects on the brain. Examples of potential treatment comparisons are described in Table 2.

**Table 1:** Examples of clinical trials testing neuroprotective properties of an anti-hypertensive or anti-diabetic drug

Drug	Class	Primary clinical use	Trials underway to evaluate the use to treat or prevent dementia or cognitive decline	Putative primary mechanism of action	Estimated completion
Nilvadipine	Calcium channel blocker	Hypertension	NILVAD Phase III trial evaluating if this calcium-channel blocker can improve cognitive function in mild-moderate Alzheimer’s disease (NCT02017340)	Beta-amyloid clearance and cortical perfusion	2017
Telmisartan versus Perindopril	ARB versus ACE inhibitor	Hypertension	SARTAN-AD Phase II head-to-head comparison of perindopril and telmisartan in Alzheimer’s patients with hypertension, using brain atrophy as an experimental surrogate marker (NCT02085265)	Beta-amyloid production and catabolism	2017
Candesartan or Losartan	ARB	Hypertension	A Phase II trial with candesartan in MCI (NCT02646982) and Losartan in Alzheimer’s (ISRCTN93682878)	Neurovascular injury, blood-flow, beta-amyloid pathways	2021 & 2017
Metformin	Biguanide	Diabetes	A Phase II in Alzheimer’s (NCT02409238) and a Phase II trial in MCI (NCT01965756) are underway	Restore insulin signaling in the brain	2017 & 2016
Pioglitazone, mini-dose	Thiazolidinedione	Diabetes but at a different dose	Phase 3 trial testing a very low-dose formulation of pioglitazone to reduce the risk MCI due to Alzheimer’s (NCT01931566)	Metabolism and inflammation	2019
Liraglutide	Incretin mimetic (GLP-1 agonist)	Diabetes	Two Phase II trials underway or recently completed in Alzheimer’s (NCT01843075; NCT01469351). A third trial is underway in aging adults at high risk of dementia (NCT02140983) and a fourth Phase III trial is underway on cognitive dysfunction in major depressive disorder or bipolar disorder (NCT02423824)	Restore insulin signaling in the brain to slow Alzheimer’s pathology	2015–2017
Exenatide (Exendin-4)	Incretin mimetic (GLP-1 agonist)	Diabetes	A Phase 2 safety trial in patients with Alzheimer’s or mild cognitive impairment with secondary outcomes of behavioral and cognitive performance, ADAS-cog and CDR, and biomarkers related to Alzheimer’s disease and dementia (NCT01255163)	Restore insulin signaling in the brain to slow Alzheimer’s pathology	2018

Examples of clinical trials underway to evaluate whether a drug approved for hypertension or diabetes could be repurposed to treat or prevent Alzheimer’s disease or cognitive impairment. Many other trials have already been completed. In all cases, the putative mechanism of action involves a direct effect on the brain rather than an indirect effect through treatment of the primary indication. Other repurposing efforts are underway with drugs approved for depression, epilepsy, and erectile dysfunction. Very few studies are designed for CER, i.e. to compare the cognitive outcomes from treatments that are clinically equivalent for their currently approved indication

*ACE* angiotensin converting enzyme inhibitor, *ARB* angiotensin receptor blocker, *MCI* mild cognitive impairment

**Table 2:** Examples of potential questions for comparative effectiveness research

Patient population	Treatment comparisons	Putative mechanisms in the brain	Clinical research
Hypertensive patients at high risk of cognitive decline	Telmisartan versus other ARBs versus centrally acting ACEi versus non-centrally acting ACEi	Polymorphisms in ACE have been linked to Alzheimer’s disease [60] but whether central ACE inhibition will protect or harm is unclear. ACEi but not ARBs might accelerate beta-amyloid pathology by blunting ACE activity on non-angiotensin pathways and by inhibiting AT2 and AT4 receptors. One ARB in particular, telmisartan, has additional activity on PPAR gamma that might protect against neurodegeneration [61]	A network meta-analysis concluded that ARBs had more benefit on cognition than ACEi drugs (adjusted effect size 0.47 +/– 0.17, *p* = 0.04) [62]. Yet hypertension management with centrally acting versus non-centrally acting ACEi was associated with 25 % slower functional decline in Alzheimer’s patients [63]. A Phase II trial is underway to compare telmisartan to perindopril in patients with comorbid hypertension and Alzheimer’s (Table 1). Additional comparisons are needed
Hypertensive patients at risk of cognitive decline	Amlodipine or nifedipine versus other DHP CCBs	Most DHP CCBs are likely to penetrate the brain except for amlodipine. Nilvadipine and nitrendipine but not amlodipine decreased beta-amyloid accumulation and blunted apoptosis in a mouse model of Alzheimer’s. DHP CCBs varied in their capacity to increase amyloid clearance from the brain [64]. Effects on the brain may vary depending on their selectivity for different calcium channels [65]	In a small trial in hypertensive patients with MCI, nilvadipine versus amlodipine slowed cognitive decline and improved cerebral blood flow despite similar effects on blood pressure [34]. Nitrendipine and nimodipine, have clinical data to suggest utility for the prevention or treatment of dementia, respectively, while nifedipine was associated with an increased risk of cognitive decline (reviewed in [64]). A Phase III trial is underway to test nilvadipine as a treatment for Alzheimer’s (Table 1)
Diabetes patients at risk of cognitive decline	Centrally penetrant versus non-penetrant GLP-1 agonists	GLP-1 agonists have been shown to protect against hippocampal synapse loss, lower beta-amyloid pathology and related damage, reduce neuroinflammation, and promote neurogenesis. While exenatide, liraglutude, and lixisenatide cross the blood–brain barrier, albiglutide and dulaglutide are large proteins unlikely to reach the brain [18]	Treatment with liraglutide blocked decline in cerebral glucose metabolism over 6 months in Alzheimer’s patients in a Phase II trial [66]. Additional trials are underway to repurpose these drugs to treat cognitive impairment (Table 1) but not to compare cognitive outcomes of CNS-penetrant versus non-penetrant GLP-1 agonists for diabetes treatment
Diabetes patients with or without comorbid dementia	Choice of drugs to minimize the risk of severe hypoglycemia	Severe hypoglycemia can trigger acute cognitive impairment and possibly accelerate long-term cognitive decline [67]. The choice of drugs used to manage diabetes may alter the risk of severe hypoglycemia in some patients	Nursing home patients with both dementia and diabetes had up to 8× higher risk of severe hypoglycemia when treated with sulphonylurea instead of insulin analogs [68]. More research is needed on how the choice of drugs alters risk in diverse patient populations

Examples of potential questions for comparative effectiveness research to examine whether the choice of clinically equivalent treatments for a given disease indication could influence the risk or rate of cognitive decline in high-risk patients

*ACEi* angiotensin converting enzyme inhibitor, *ARB* angiotensin receptor blocker, *CCB* calcium channel blocker, *DHP* dihydropyridine

The examples provided are not intended to comprehensively review the available data but rather to highlight the many ways through which common clinical care decisions might slow cognitive decline or reduce the risk of dementia. More research is needed to guide clinical care. Below, we recommend five general approaches to enable or bolster research that could create an evidence base to guide clinical care to mitigate the risk of cognitive decline or dementia in vulnerable patients.

## Main text

### Recommendation 1: researchers should utilize complementary study designs that incorporate patients at high risk for cognitive decline

The study of real-world populations is central to CER yet particularly challenging for research on cognitive decline. Patients with advanced age or comorbidities, who are highly vulnerable to cognitive decline, are rarely included in research (e.g. [32]). Even in geriatrics, cognitively impaired patients are rarely recruited for clinical research on other conditions [33] and those that develop impairment during the trial are likely to drop out before their outcome can be assessed [14]. Most of the clinical research that has been done on other health conditions cannot therefore accurately inform how those treatments affect cognitive decline and dementia risk even if those outcomes were recorded.

Another challenge is the heterogeneous nature of dementia, as described above. The treatments that reduce the risk of decline and the trials capable of detecting a benefit may succeed in one population but not another. To overcome these challenges, a combination of study designs with complementary strengths and weaknesses that include patients at high risk of cognitive decline is needed.

Pragmatic Phase III randomized controlled trials (RCTs) are most likely to influence clinical care but there are limits on our ability to fund and carry out multiple randomized head-to-head comparisons with sufficient power and follow-up. Short-term RCTs rarely have sufficient power to detect clinically meaningful change in cognitive decline and related function but shorter trials can use biomarker endpoints to validate the putative disease-modifying effects of treatments. For example, a pilot trial reported that hypertension management with nilvadipine versus amlodipine might improve cerebral blood flow in patients with MCI despite similar effects on blood pressure [34]. Currently, a trial is underway at the Sunnybrook Research Institute in Canada to compare hypertension management with telmisartan versus perindopril in patients with comorbid Alzheimer’s, looking at global brain atrophy over one year (Table 1). Although these biomarkers are not validated as surrogate markers, these exploratory trials can raise confidence for larger and longer trials on patient-centered outcomes.

Observational study designs are essential tools for comparative effectiveness research (CER) [35] and dementia prevention research [36, 37] that provide windows into real-world heterogeneous patient populations. These studies are at high risk of confounding by indication and other bias but robust associations with cognitive decline can still inform hypotheses and guide clinical trial design.

There are several examples of the use of electronic health records and related databases for exploratory questions. The use of angiotensin receptor blockers (ARBs) compared to other cardiovascular drugs was associated with a 24 % lower risk of incident dementia in the US Veteran Affairs database [38]. The use of proton pump inhibitors was associated with an increased risk of all-cause dementia (HR 1.33; 95 % CI 1.04–1.83) and a 44 % increased risk of Alzheimer’s disease from a German database on primary care patients (HR 1.44; 95 % CI 1.01–2.06) [25]. These exploratory associations should be followed up with additional observational studies to confirm the association and inform the design of RCTs.

### Recommendation 2: incorporate cognitive assessment of high-risk individuals into routine clinical evaluations and electronic health records

In order to learn whether specific clinical care decisions influence cognitive decline, better recognition and reporting of cognitive function is needed in clinical settings particularly for patients at risk of cognitive decline because of comorbidities, frailty, age, genetics, or family history. Between 27 % and 81 % of cases of cognitive impairment are not currently recognized in primary care [39]. When a patient is diagnosed, they often have fairly advanced impairment with little if any objective data showing the trajectory of development of their impairment over time.

Some groups have recommended widespread screening for MCI and/or dementia (e.g. [39–41]) while others have called for research to prove that such screening improves patient outcomes [42]. While this debate continues, a parallel consideration is annual cognitive testing that informs clinical care even if it not used for diagnostic screening.

Objectively measured cognitive ability has been recommended as an important variable to inform the clinical care of geriatric patients with diseases like diabetes [43], cancer [44], and heart failure [45]. Routine cognitive evaluations can identify patients who might need additional medication reminders or other treatments for anxiety, depression, insomnia, polypharmacy, alcoholism, or drug abuse. They can also create a longitudinal record to help inform prognosis in later years if the patient or their family reports subjective cognitive impairment. Importantly for this discussion, regular cognitive evaluations can create a longitudinal record of cognitive assessments in real-world patient populations which could inform CER on which treatments associate with better or worse cognitive trajectories in which patients. A variety of neuropsychological tests are suitable for general practice, with strengths and weaknesses reviewed elsewhere [39, 40].

### Recommendation 3: in comparative effectiveness trials, incorporate outcomes on dementia incidence, cognitive decline, or neurodegeneration

One strategy to increase the feasibility of CER on dementia risk or cognitive decline is to embed secondary outcomes in trials designed for other conditions. Several examples demonstrate that this can be done successfully. A sub-study of the ACCORD T2DM trial compared the effects of intensive versus standard blood glucose control on cognitive decline and brain atrophy [19]. The SPRINT trial, comparing intensive versus less intensive control of systolic blood pressure, was stopped early due to a significant reduction in the primary cardiovascular outcome [46]. For modest incremental cost, the trial also included assessment of all-cause dementia incidence, global cognitive decline, and MRI-measured changes in brain structure [47], with results yet to be reported.

A major concern with this strategy is outcome ascertainment bias [14]. Unlike major cardiovascular endpoints, cognitive outcomes cannot be readily captured from medical databases for patients who fail to return to the research clinic. Trial methods can be adapted, however, as achieved in the Ginkgo Evaluation of Memory Study [48]. Another issue is that trials may be stopped early because of emergence of clear differences in the primary outcome (e.g. [46]). Extended follow-up may be needed to ascertain differences in cognitive outcomes that may take longer to emerge.

More recently in the Alzheimer's disease  field, there has been a change in emphasis to “slow progression” of the disease to “prevent” or “delay” the onset of the disease itself. This implies the need to rethink traditional study designs, including whether cognitive endpoints are the most suitable ones for such studies. As such studies tend to be more like epidemiological studies, “time to event” may be more suitable than a cognitive test, particularly since cognitive changes in a “prevention” setting tend to be small/subtle (much less the functional changes, which are even more subtle), though it is not yet clear which outcomes are best for such studies.

### Recommendation 4: develop, validate, and standardize practical and acceptable methods for frequent assessment of cognition and function

Cognitive function varies on a daily, even hourly basis due to sleep impairment, alcohol or nicotine use, emotional or physical status, and other variables. Similar variation exists for quality of life and activities of daily living. This variability weakens the power to detect meaningful change with infrequent, intermittent assessments.

Neuropsychiatric testing deployed on consumer-devices has the potential to lower costs and increase the capacity of frequent assessment, particularly with data passively collected from routine patient behavior [49]. For example, a patient’s conversations might be recorded and processed with automated speech-analysis software to detect the changes in verbal fluency and syntactic complexity that may be symptomatic of MCI (reviewed in [49]). An unobtrusive home-monitoring system can measure parameters of activity such as gait deficits, stride variability, and functional activities of daily living [50]. A different approach adopted by Akili Interactive Labs, Inc. is to design assays that entertain patients and thereby encourage more frequent assessment.

These technologies may provide statistical power that is not possible with current clinical instruments for activities of daily living or cognitive ability. For example, an estimated 80 % fewer patients would be required to detect a change in behavior using the unobtrusive home monitoring system mentioned above compared to conventional annual neuropsychological testing [51]. It may also provide the basis for collecting the “real-world” evidence being sought by the 21st Century Cures Act.

The rapid evolution of technology creates opportunities but also challenges. The ways in which data are collected, anonymized, and aggregated will need to be standardized before becoming widely adopted in clinical research. Developers will need to define and demonstrate the context of use for a given assessment tool (e.g. prognosis, diagnosis, rate of decline) and the applicability of the test to different populations (e.g. the influence of language, culture, and disease state). As with other types of ever-expanding data collected on individual citizens, careful attention will be needed to guard against risks to privacy, individual rights, and ethical abuse.

### Recommendation 5: explore innovations in policy and funding to encourage healthcare providers and pharmaceutical companies to engage in comparative effectiveness research

The scope of CER on cognitive decline will be severely limited without investment and participation by organizations outside of academic and government settings. In theory, a drug would have a market advantage if its use was shown to yield a lower risk of long-term cognitive decline. In practice, however, companies have little commercial incentive to pursue either CER [52] or repurposing of  products for high-risk indications like dementia [53]. Policy changes may help to incentive industry, for example with an increased duration of data exclusivity after approval of a new indication [53]. Alternatively, the need for industry investment might be reduced by strategies to lower the cost of CER and repurposing, for example with preliminary or staged approval for a new indication followed by post-approval pharmacovigilance to confirm safety and efficacy [53].

Healthcare providers may similarly lack incentive to participate in CER, which limits the potential to understand real-world patient care using electronic medical records and related databases [52]. Providers need resources, reimbursement, and training to evaluate patient cognition and to understand the potential relationships between clinical care and cognitive decline. They also need compelling evidence that cognitive assessment improves patient care without slowing provider productivity or raising costs. Improved communication between researchers and frontline healthcare providers could also improve the clinical relevance of CER experimental design [54].

## Conclusions

Over the past decade, CER has received more and more attention in the United States and elsewhere. Patients, families, healthcare providers, and payers increasingly ask which treatments have the best benefit versus harm ratio for a given patient. As this research advances, we recommend increased assessment of cognitive decline and the risk of dementia or MCI in CER studies. These outcomes are central to patient interests, quality of life, medical care, and societal costs. They are also likely to be influenced in profound ways by routine clinical care decisions.

Many challenges exist in the design, implementation, and funding of such research. Nevertheless, creative solutions exist and resources for CER (e.g. PCORI) may be leveraged to meet this need. Stakeholders across the spectrum of patients, providers, payers, researchers, and regulators can all play a role in accelerating the development of an evidence base so that clinicians and patients can learn whether the management of their existing conditions can influence the risk of cognitive decline and dementia.

CER in older people is a particular challenge because the vulnerabilities caused by aging often lead to comorbid  chronic and acute diseases and ailments such as frailty that impair quality of life and function. Indeed, frailty and the accumulation of health deficits with old age have themselves been linked to a higher risk of dementia [23, 55]. In these vulnerable populations, it can be challenging if not impossible to compare the full breadth of clinical benefit versus harm of diverse treatment options. Some therapeutics, however, may alter specific aspects of aging biology either through direct effects [56] or indirect effects via the management of comorbidities linked to accelerated aging (e.g. [57–59]). In order to assess which therapies have the best harm versus benefit ratio in older people, there is a strong need for defined and validated independent measures of elements of aging that affect morbidity that could be evaluated more efficiently than the incidence of numerous single clinical diseases. The rate of decline in certain domains of cognitive function (e.g. language, executive function) could be one of several components of such a screen for independent measures of morbidity associated with aging.

## Box 1: Questions to consider before funding studies of a given comparative effectiveness question, particularly a Phase III clinical trial

1. Will the results have a chance to affect clinical practice? Data on the risk for cognitive decline or dementia will have a greater influence on clinical practice if the treatments being compared are clinically equivalent for their primary indication and identical to the treatment regimen used for the primary indication. In addition, clinical practice sometimes evolves before a given comparative effectiveness study has been completed. Stakeholders should evaluate whether drugs in development or other sources of change might reduce the clinical relevance of the study by the time it has completed.
2. If the results do affect clinical practice, how many patients will be protected from cognitive decline? Computational models can help to predict the magnitude of benefit that might be achieved at a population level.
3. What preliminary evidence predicts that the clinical comparison will influence cognitive decline? Does it include diverse and complementary evidence from laboratory experiments in different model system, observations of real-world patients, and experimental trials on short-term biomarker outcomes?
4. Can the study be successfully completed given challenges in patient recruitment and retention? Will the results from the study, whether positive or negative, be conclusive?
5. Is the study design appropriate? Will the results have external validity for real-world clinical populations? Will the results have strong internal validity with a low risk of residual confounding and bias? To address both these issues, a combination of study designs may be needed with complementary strengths and weaknesses.
6. Could short-term investment in infrastructure or assay development lead to faster and more accurate comparative effectiveness research? The capacity for comparative effectiveness research is rapidly evolving. Investments to help design these infrastructures to address cognitive impairment and dementia risk could improve the reliability and validity of subsequent research.

## Abbreviations

CER, Comparative effectiveness research; MCI, Mild cognitive impairment; PCORI, Patient Centered Outcomes Research Institute

## References

[1] Prince M, Wimo A, Guerchet M, Ali GC, Wu YT, Prina M, et al. The global impact of dementia: an analysis of prevalence, incidence, cost and trends. World Alzheimer Report 2015 [Internet]. 2015. http://www.alz.co.uk/research/WorldAlzheimerReport2015.pdf. Accessed May 2016.

[2] Lin PJ, Neumann PJ (2013). The economics of mild cognitive impairment. Alzheimers Dement.

[3] Teng E, Tassniyom K, Lu PH (2012). Reduced quality-of-life ratings in mild cognitive impairment: analyses of subject and informant responses. Am J Geriatr Psychiatry.

[4] Callahan KE, Lovato JF, Miller ME, Easterling D, Snitz B, Williamson JD (2015). Associations between mild cognitive impairment and hospitalization and readmission. J Am Geriatr Soc.

[5] Medicine I, Blazer DG, Yaffe K, Liverman CT (2015). Cognitive Aging: Progress in Understanding and Opportunities for Action.

[6] FDA. In: FDA, editor. Targeted Drug Development: Why Are Many Diseases Lagging Behind? 2015. The report referenes was downloaded from theFDA.gov website at http://www.fda.gov/downloads/AboutFDA/ReportsManualsForms/Reports/UCM454996.pdf. Accessed April 2016.

[7] Launer LJ (2015). Preventing Alzheimer’s disease is difficult. Lancet Neurol.

[8] Tang EY, Harrison SL, Errington L, Gordon MF, Visser PJ, Novak G (2015). Current developments in dementia risk prediction modelling: an updated systematic review. PLoS One.

[9] White LR, Edland SD, Hemmy LS, Montine KS, Zarow C, Sonnen JA (2016). Neuropathologic comorbidity and cognitive impairment in the Nun and Honolulu-Asia Aging Studies. Neurology.

[10] Rouch L, Cestac P, Hanon O, Cool C, Helmer C, Bouhanick B (2015). Antihypertensive drugs, prevention of cognitive decline and dementia: a systematic review of observational studies, randomized controlled trials and meta-analyses, with discussion of potential mechanisms. CNS Drugs.

[11] Forette F, Seux ML, Staessen JA, Thijs L, Babarskiene MR, Babeanu S (2002). The prevention of dementia with antihypertensive treatment: new evidence from the Systolic Hypertension in Europe (Syst-Eur) study. Arch Intern Med.

[12] Tzourio C, Anderson C, Chapman N, Woodward M, Neal B, MacMahon S (2003). Effects of blood pressure lowering with perindopril and indapamide therapy on dementia and cognitive decline in patients with cerebrovascular disease. Arch Intern Med.

[13] McGuinness B, Todd S, Passmore P, Bullock R (2009). Blood pressure lowering in patients without prior cerebrovascular disease for prevention of cognitive impairment and dementia. Cochrane Database Syst Rev..

[14] Di Bari M, Pahor M, Franse LV, Shorr RI, Wan JY, Ferrucci L (2001). Dementia and disability outcomes in large hypertension trials: lessons learned from the systolic hypertension in the elderly program (SHEP) trial. Am J Epidemiol.

[15] Jacobs V, Cutler MJ, Day JD, Bunch TJ (2015). Atrial fibrillation and dementia. Trends Cardiovasc Med.

[16] de Bruijn RF, Heeringa J, Wolters FJ, Franco OH, Stricker BH, Hofman A, et al. Association between atrial fibrillation and dementia in the general population. JAMA Neurol. 2015:1–7. doi: 10.1001/jamaneurol.2015.2161. 10.1001/jamaneurol.2015.2161 26389654

[17] Vagelatos NT, Eslick GD (2013). Type 2 diabetes as a risk factor for Alzheimer’s disease: the confounders, interactions, and neuropathology associated with this relationship. Epidemiol Rev..

[18] Sebastiao I, Candeias E, Santos MS, de Oliveira CR, Moreira PI, Duarte AI (2014). Insulin as a bridge between type 2 diabetes and alzheimer disease - how anti-diabetics could be a solution for dementia. Front Endocrinol (Lausanne).

[19] Launer LJ, Miller ME, Williamson JD, Lazar RM, Gerstein HC, Murray AM (2011). Effects of intensive glucose lowering on brain structure and function in people with type 2 diabetes (ACCORD MIND): a randomised open-label substudy. Lancet Neurol.

[20] Yaffe K, Falvey CM, Hoang T (2014). Connections between sleep and cognition in older adults. Lancet Neurol.

[21] Cermakova P, Eriksdotter M, Lund LH, Winblad B, Religa P, Religa D (2015). Heart failure and Alzheimer’s disease. J Intern Med.

[22] Dodd JW (2015). Lung disease as a determinant of cognitive decline and dementia. Alzheimers Res Ther.

[23] Armstrong JJ, Mitnitski A, Andrew MK, Launer LJ, White LR, Rockwood K (2015). Cumulative impact of health deficits, social vulnerabilities, and protective factors on cognitive dynamics in late life: a multistate modeling approach. Alzheimers Res Ther.

[24] Gray SL, Anderson ML, Dublin S, Hanlon JT, Hubbard R, Walker R (2015). Cumulative use of strong anticholinergics and incident dementia: a prospective cohort study. JAMA Intern Med.

[25] Gomm W, von Holt K, Thome F, Broich K, Maier W, Fink A (2016). Association of proton pump inhibitors with risk of dementia: a pharmacoepidemiological claims data analysis. JAMA Neurol.

[26] Fong TG, Davis D, Growdon ME, Albuquerque A, Inouye SK (2015). The interface between delirium and dementia in elderly adults. Lancet Neurol.

[27] Avidan MS, Evers AS (2016). The fallacy of persistent postoperative cognitive decline. Anesthesiology.

[28] Norton S, Matthews FE, Barnes DE, Yaffe K, Brayne C (2014). Potential for primary prevention of Alzheimer’s disease: an analysis of population-based data. Lancet Neurol.

[29] Barnard ND, Bush AI, Ceccarelli A, Cooper J, de Jager CA, Erickson KI (2014). Dietary and lifestyle guidelines for the prevention of Alzheimer’s disease. Neurobiol Aging..

[30] Martinez-Lapiscina EH, Clavero P, Toledo E, San Julian B, Sanchez-Tainta A, Corella D (2013). Virgin olive oil supplementation and long-term cognition: the PREDIMED-NAVARRA randomized, trial. J Nutr Health Aging.

[31] Ngandu T, Lehtisalo J, Solomon A, Levalahti E, Ahtiluoto S, Antikainen R (2015). A 2 year multidomain intervention of diet, exercise, cognitive training, and vascular risk monitoring versus control to prevent cognitive decline in at-risk elderly people (FINGER): a randomised controlled trial. Lancet.

[32] Tinetti ME (2014). The gap between clinical trials and the real world: extrapolating treatment effects from younger to older adults. JAMA Intern Med.

[33] Taylor JS, DeMers SM, Vig EK, Borson S (2012). The disappearing subject: exclusion of people with cognitive impairment and dementia from geriatrics research. J Am Geriatr Soc.

[34] Hanyu H, Hirao K, Shimizu S, Iwamoto T, Koizumi K, Abe K (2007). Favourable effects of nilvadipine on cognitive function and regional cerebral blood flow on SPECT in hypertensive patients with mild cognitive impairment. Nucl Med Commun.

[35] Velentgas P, Dreyer NA, Nourjah P, Smith SR, Torchia MM (2013). Developing a Protocol for Observational Comparative Effectiveness Research: A User’s Guide.

[36] Dacks PA, Andrieu S, Blacker D, Carman AJ, Green AM, Grodstein F (2014). Dementia Prevention: optimizing the use of observational data for personal, clinical, and public health decision-making. J Prev Alzheimers Dis.

[37] Solomon A, Mangialasche F, Richard E, Andrieu S, Bennett DA, Breteler M (2014). Advances in the prevention of Alzheimer’s disease and dementia. J Intern Med.

[38] Li NC, Lee A, Whitmer RA, Kivipelto M, Lawler E, Kazis LE (2010). Use of angiotensin receptor blockers and risk of dementia in a predominantly male population: prospective cohort analysis. BMJ..

[39] Cordell CB, Borson S, Boustani M, Chodosh J, Reuben D, Verghese J (2013). Alzheimer’s Association recommendations for operationalizing the detection of cognitive impairment during the Medicare Annual Wellness Visit in a primary care setting. Alzheimers Dement.

[40] Morley JE, Morris JC, Berg-Weger M, Borson S, Carpenter BD, Del Campo N (2015). Brain Health: The Importance of Recognizing Cognitive Impairment: An IAGG Consensus Conference. J Am Med Dir Assoc.

[41] DIAGNOSIS TGSOAWOCIDAE. Report and Recommendations. 2015.

[42] Lin JS, O’Connor E, Rossom RC, Perdue LA, Burda BU, Thompson M (2013). Screening for Cognitive Impairment in Older Adults: An Evidence Update for the US Preventive Services Task Force [Internet]. U.S. Preventive Services Task Force Evidence Syntheses, formerly Systematic Evidence Reviews.

[43] Moreno G, Mangione CM, Kimbro L, Vaisberg E, American Geriatrics Society Expert Panel on Care of Older Adults with Diabetes M (2013). Guidelines abstracted from the American Geriatrics Society Guidelines for Improving the Care of Older Adults with Diabetes Mellitus: 2013 update. J Am Geriatr Soc.

[44] Lange M, Rigal O, Clarisse B, Giffard B, Sevin E, Barillet M (2014). Cognitive dysfunctions in elderly cancer patients: a new challenge for oncologists. Cancer Treat Rev.

[45] Cameron J, Pressler SJ, Ski CF, Thompson DR (2014). Cognitive impairment in heart failure: towards a consensus on screening. Eur J Heart Fail.

[46] Group SR, Wright JT, Williamson JD, Whelton PK, Snyder JK, Sink KM (2015). A randomized trial of intensive versus standard blood-pressure control. N Engl J Med.

[47] Ambrosius WT, Sink KM, Foy CG, Berlowitz DR, Cheung AK, Cushman WC (2014). The design and rationale of a multicenter clinical trial comparing two strategies for control of systolic blood pressure: the Systolic Blood Pressure Intervention Trial (SPRINT). Clin Trials.

[48] Williamson JD, editor. The Ginkgo in Evaluation of Memory (GEM) study: Lessons learned for planning future dementia primary prevention trials. Alzheimers Dement. 2009;(5):P144. doi:10.1016/j.jalz.2009.05.492.

[49] Laske C, Sohrabi HR, Frost SM, Lopez-de-Ipina K, Garrard P, Buscema M (2015). Innovative diagnostic tools for early detection of Alzheimer’s disease. Alzheimers Dement.

[50] Lyons BE, Austin D, Seelye A, Petersen J, Yeargers J, Riley T (2015). Pervasive computing technologies to continuously assess Alzheimer’s disease progression and intervention efficacy. Front Aging Neurosci..

[51] Dodge HH, Zhu J, Mattek NC, Austin D, Kornfeld J, Kaye JA (2015). Use of high-frequency in-home monitoring data may reduce sample sizes needed in clinical trials. PLoS One.

[52] Friedly JL, Bauer Z, Comstock BA, DiMango E, Ferrara A, Huang SS (2014). Challenges conducting comparative effectiveness research: the Clinical and Health Outcomes Initiative in Comparative Effectiveness (CHOICE) experience. Dovepress.

[53] Shineman DW, Alam J, Anderson M, Black SE, Carman AJ, Cummings JL (2014). Overcoming obstacles to repurposing for neurodegenerative disease. Ann Clin Transl Neurol.

[54] Pierce BA, Chesney MA, Witt CM, Berman BM (2012). Physician perspectives on comparative effectiveness research: implications for practice-based evidence. Glob Adv Health Med.

[55] Searle SD, Rockwood K (2015). Frailty and the risk of cognitive impairment. Alzheimers Res Ther.

[56] Kirkland JL (2016). Translating the science of aging into therapeutic interventions. Cold Spring Harb Perspect Med.

[57] Geersing GJ, de Groot JA, Reitsma JB, Hoes AW, Rutten FH (2015). The impending epidemic of chronic cardiopulmonary disease and multimorbidity: the need for new research approaches to guide daily practice. Chest.

[58] Morley JE (2008). Diabetes and aging: epidemiologic overview. Clin Geriatr Med.

[59] Lohr JB, Palmer BW, Eidt CA, Aailaboyina S, Mausbach BT, Wolkowitz OM (2015). Is post-traumatic stress disorder associated with premature senescence? A review of the literature. Am J Geriatr Psychiatry.

[60] Kauwe JS, Bailey MH, Ridge PG, Perry R, Wadsworth ME, Hoyt KL (2014). Genome-wide association study of CSF levels of 59 Alzheimer’s disease candidate proteins: significant associations with proteins involved in amyloid processing and inflammation. PLoS Genet.

[61] Fournier A, Oprisiu-Fournier R, Serot JM, Godefroy O, Achard JM, Faure S (2009). Prevention of dementia by antihypertensive drugs: how AT1-receptor-blockers and dihydropyridines better prevent dementia in hypertensive patients than thiazides and ACE-inhibitors. Expert Rev Neurother.

[62] Levi Marpillat N, Macquin-Mavier I, Tropeano AI, Bachoud-Levi AC, Maison P (2013). Antihypertensive classes, cognitive decline and incidence of dementia: a network meta-analysis. J Hypertens.

[63] O'Caoimh R, Healy L, Gao Y, Svendrovski A, Kerins DM, Eustace J (2014). Effects of centrally acting angiotensin converting enzyme inhibitors on functional decline in patients with Alzheimer’s disease. J Alzheimers Dis.

[64] Peters J, Booth A, Peters R (2015). Potential for specific dihydropyridine calcium channel blockers to have a positive impact on cognitive function in humans: a systematic review. Ther Adv Chronic Dis.

[65] Nimmrich V, Eckert A (2013). Calcium channel blockers and dementia. Br J Pharmacol.

[66] Gejl M, Gjedde A, Egefjord L, Moller A, Hansen S, Vang K (2015). No Decline of Brain Glucose Metabolism in Alzheimer’s Disease Patients Treated with Liraglutide.

[67] Frier BM (2014). Hypoglycaemia in diabetes mellitus: epidemiology and clinical implications. Nat Rev Endocrinol.

[68] Abbatecola AM, Bo M, Barbagallo M, Incalzi RA, Pilotto A, Bellelli G (2015). Severe hypoglycemia is associated with antidiabetic oral treatment compared with insulin analogs in nursing home patients with type 2 diabetes and dementia: results from the DIMORA study. J Am Med Dir Assoc.

